# Best analgesia control in pancreatic adenocarcinoma study: justification and feasibility of a randomised trial of early EUS-CPN versus standard care—a prospective observational study (The BAC-PAC study)

**DOI:** 10.1038/s44276-023-00013-x

**Published:** 2023-09-18

**Authors:** Andreas I. Koulouris, Adam P. Wagner, Allan Clark, Leo Alexandre

**Affiliations:** 1 Norwich Medical School, University of East Anglia, Norwich Research Park, Norwich, Norfolk, NR4 7TJ UK; 2 Norfolk and Norwich University Hospital, Norwich, Colney Lane, NR4 7UY UK; 3 NIHR Applied Research Collaboration (ARC) East of England (EoE), Douglas House, 18 Trumpington Road, CB2 8AH Cambridge, UK

## Abstract

**Introduction:**

Limited and conflicting trial data is available on the efficacy of Endoscopic Ultrasound-guided Coeliac Plexus Neurolysis (EUS-CPN). This study aimed to assess the feasibility, justification and to inform design considerations of a randomised trial of early EUS-CPN versus standard care.

**Methods:**

This was a questionnaire-based prospective observational study of patients with inoperable pancreatic adenocarcinoma who were self-reporting their performance status, pain levels, analgesic use, quality of life (QoL) and healthcare resource use, on a monthly basis.

**Results:**

Over a total period of twelve months 143 patients were screened for eligibility, of which 56 met the criteria. In total, 12 (21%) patients were recruited. The median survival from the first record of pain was 5.2 (IQR 2.46–5.9) months. In total, 80% of the questionnaires were completed. The median Visual Analogue Score for pain was 2.6 (0.8–5.1) and the median daily morphine dose was 36 (20–48) mg.

**Discussion:**

Recruitment rates remained low throughout this study. Despite these limitations, overall, this study supports the justification of trial administering endoscopic analgesia. However, uncertainties remain with regards to its feasibility. In a future trial, data collection procedures need to minimise burden to patients. Further observational research with a larger sample size, longer follow-up and refined procedures is required.

## Introduction

Pancreatic cancer has the worst prognosis of any cancer, with only 21% of the patients surviving beyond a year [1]. Patients can be divided in those who are surgical candidates, those eligible for chemotherapy and those eligible only for supportive and end of life care. In patients with unresectable disease, chemotherapy can prolong life, achieving a median survival of 11.1 months (95% CI, 9.0 to 13.1) [2]. Most patients who only receive supportive care survive for two to six months [2, 3]. Previous studies have shown that 58–78% of patients develop abdominal pain of pancreatic origin during the course of their disease [4, 5]. Over time, opiate doses required for pain control escalate from a mean of 55.9 mg (SD 53.8) at diagnosis to 162.8 mg (SD 131.6) towards the end of life [4, 6–9]. These doses can lead to serious side-effects such as gastroparesis, constipation, lethargy and cognitive decline [6] and which can severely impact their quality of life during their typically short survival times.

Endoscopic Ultrasound-guided Coeliac Plexus Neurolysis (EUS-CPN) is a minimally invasive procedure which causes chemical ablation of the coeliac ganglia and disrupts efferent pain signalling [10]. EUS-CPN is usually reserved as a second line analgesic option when opioids have failed to control pain. In total, 17 clinical studies have assessed the efficacy and safety of EUS-CPN in the past 25 years [11]. Of those, ten were phase II trials which compared pain levels in the same participants before and after the procedure [12–21]. Five studies had two treatments arms, each one offering variations of the EUS-CPN technique, differing by injection location (central vs bilateral vs within the ganglia) or the volume or type of neurolytic agent or method (alcohol vs phenol vs radiofrequency ablation) [22–26]. Only one phase III randomised controlled trial by Wyse et al. has investigated the efficacy of standard EUS-CPN (with absolute alcohol used as the neurolytic agent injected around the ganglia) versus opioids alone [27]. The included patients had locally advanced cancer and each arm included 54 patients. Between baseline and three months, the control group mean pain score increased by 12% (95% CI, −19% to 36%), in contrast to the EUS-CPN group where it decreased by 49% (95% CI, 38% to 61%). The difference in the mean percentage change of pain scores between groups at three months showed a greater drop of 60.7% (95% CI, 25.5% to 86.6%, *P* = 0.01) in the EUS-CPN group.

Differences were also observed in the opioid consumption between arms. The control group used a mean of 36 (SD 62) mg of opioids at baseline. This figure increased by 54 mg (95% CI, 20 to 96) from baseline to month one and continued to increase over time, resulting in the mean dose increasing by 100 mg (95% CI, 49 to 180) from baseline to month three. The intervention group started from 42 (SD 71) and increased consumption by 53 mg (95% CI, 28 to 89) at month one but then opioid requirements stabilised; consequently, the increase from baseline to three months was only 50 mg (95% CI, 28 to 79). The mean opioid doses at each time point are not reported but it can be inferred that the control group was using approximately 90 mg at month one and 136 mg at month three, whilst the intervention group was using 94 mg at month one and 91 mg at month three. Mean opioid consumption was 49 mg less (95% CI, −7.0 to 127.0) at 3 months in the EUS-CPN group, but this difference was not statistically significant (*p* = 0.10). This trial only included patients with pain at diagnosis who had locally advanced disease: consequently, recruitment was limited to a mere 10% of the overall cohort with inoperable pancreatic cancer.

Kanno et al. conducted a clinical trial of 48 patients with advanced pancreatic cancer who were randomised to EUS-CPN versus morphine [28]. In total, 58% of the participants had metastatic disease, but their outcomes were not reported separately to those with locally advanced disease. At four weeks, the mean pain score for the EUS-CPN group was 1.3 (SD 1.3) versus 2.3 (SD 2.3) among controls and but this difference was not statistically significant (*p* = 0.10). Mean opioid dose in the EUS-CPN group was 62 mg (SD 2.5) versus 35 mg (SD 2.0) in the controls (*p* = 0.14). Inferences from this study were limited by the high number of drop-outs rate (7 in the intervention and 3 in the control group) and the small sample size.

Furthermore, there has been limited exploration of quality of life (QoL) impacts and associated costs to the health system (in the UK, from the perspective of the National Health System (NHS) and personal social services (PSS)), of the two different approaches to pain management. If early EUS-CPN improves pain control and keeps opioids to a lower level, its recipients are less likely to have opioid toxicity and maintain QoL for longer. In contrast, patients treated with opioids alone may be more prone to faster decline due to side-effects (lethargy, nausea etc) and therefore experience greater QoL impairment. However, using EUS-CPN as a first line analgesic measure, and therefore with a wider group of patients, is likely more costly (at least in terms of upfront costs) compared to EUS-CPN only on demand. Therefore, a cost-utility analysis, can be used to compare QoL, and the associated costs, of the alternatives to establish the most cost-effectiveness approach.

The optimal time to deliver EUS-CPN is unclear: in particular, whether it is better delivered early (as soon as pain develops) or whether it should be reserved for those with opioid-refractory pain or opioid toxicity. It is plausible that offering early EUS-CPN may prevent opioid dose escalation and preserve QoL for longer. NICE, in its latest position statement, supports the conduct of a randomised trial of early EUS-CPN versus standard care (i.e. opioids +/− on demand EUS-CPN) [29]. However, further preparatory research is necessary to first establish the optimal trial design, and, secondly, demonstrate the feasibility of such a trial.

The overarching aim of the BAC-PAC study was to determine the rationale, feasibility and refine the design considerations of a future trial of early EUS-CPN versus standard care. To inform these aims, the following objectives were set: (1) medical performance status at the onset of pain; the proportion of all patients with inoperable pancreatic cancer who are potentially fit enough for an EUS-CPN will reflect the magnitude of this clinical problem and plan the number of centres required for a future trial, (2) median survival after pain first develops; survival after pain onset has to be sufficient to justify offering an intervention and having the time to assess the effect of it in the context of a clinical trial, (3) characteristics of participants versus those who refused participation; a comparison of demographic and clinical characteristics between patients who accept and those who decline participation assesses generalizability of our estimates to the total population eligible for the study, (4) The QoL of carers of pancreatic cancer patients at monthly intervals; if QoL is severely impaired, this may further justify assessing EUS-CPN to improve QoL in patients which could consequently enhance that of their carers, (5) the proportions of patients who complete questionnaires on: medical performance status, QoL, pain scores and health resource use to assess the feasibility of patient-reporting outcomes in the context of a randomised controlled trial, including a health economic analysis, (6) time from diagnosis to first opioid prescription; this will inform the timescales for reviewing and approaching patients for randomization into a future trial, (7) the descriptive statistics of the QoL scores, abdominal pain score and opioid doses; this will aid estimating sample sizes for a future trial. Objectives 1 to 4 are intended to provide evidence to justify a future trial of early EUS-CPN; 5 to 7 are intended to inform the feasibility and planning of a future trial.

## Methods

### Study design

**Fig. 1:**
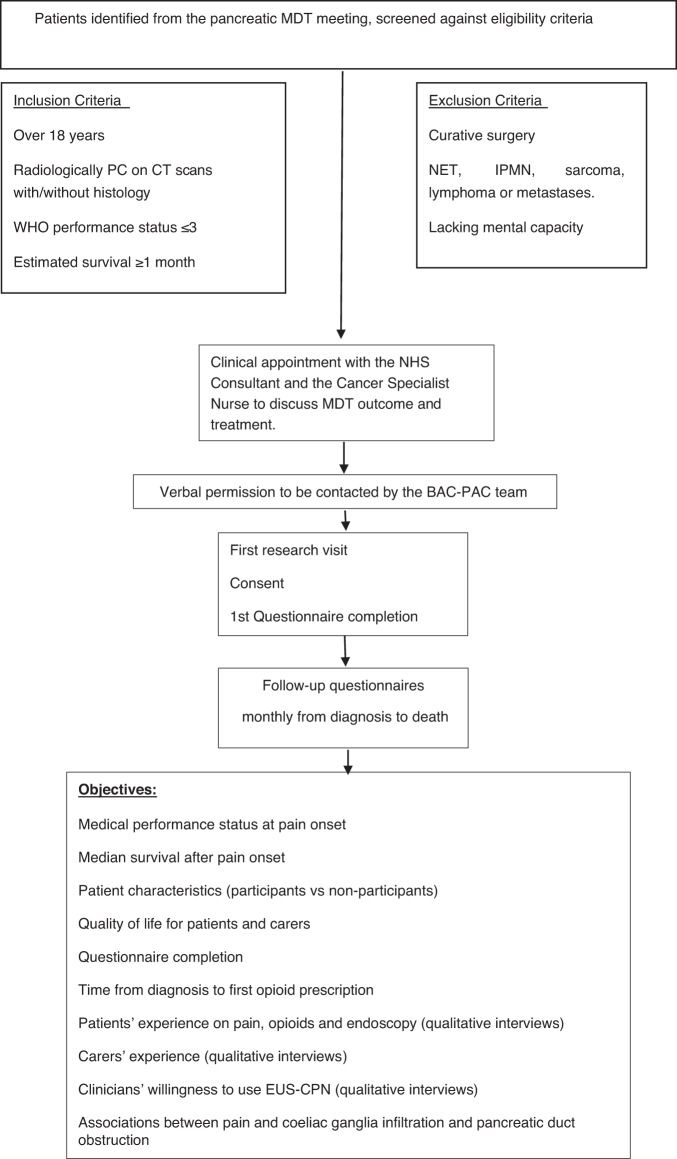
Summary of the Best Analgesia Control in Pancreatic Adenocarcinoma (BAC-PAC) study design.

Patients with inoperable pancreatic cancer were identified through the weekly multi-disciplinary pancreatic cancer team meetings. Patients were monitored for pancreatic pain and other relevant clinical outcomes from diagnosis to death, through monthly self-completed questionnaires. In addition, their primary (unpaid) caregiver, usually partner or other close relative, was asked to complete QoL questionnaires at the same time points. The study design is summarised in Fig. 1. The study was approved by the East Midlands- Leicester Central Research Ethics Committee (reference number: 19/EM/0230).

### Research setting and delivery

This study was conducted at the Norfolk and Norwich University NHS Foundation Trust (NNUH) and the James Paget University Hospital (JPUH). The research was hosted within the gastroenterology departments at each site. The University of East Anglia (UEA) sponsored the study. The study was adopted by the UK Cancer Research Network [30]. The recruitment period in NNUH lasted for a total of twelve months in two instalments: from 11th of October 2019 to 6th of March 2020 and 22nd of July 2020 to 28th of February 2021. The recruitment in JPUH was open from 2nd of September 2019 to 6th of March 2020. The gap in the recruitment period was due to the COVID-19 pandemic when the Heath Research Authority suspended all the non-essential research and clinical academic personnel were deployed to support clinical services.

### Study participants

Patients were included in the study if they: (1) were over 18 years of age, (2) had either radiologically and/or cytologically proven diagnosis of inoperable pancreatic adenocarcinoma, confirmed by the pancreatic Multi-Disciplinary Team, (3) were undergoing chemotherapy or palliative care alone, (4) had East Co-operative Oncology Group (ECOG) performance status ≤3 and 5. had estimated survival time since patient informed of diagnosis >1 month. Exclusions were applied to patients who: (1) were undergoing potentially curative surgery, (2) are suffering from non-adenocarcinoma neoplasms (neuroendocrine tumours (NETS), Intra-ductal Papillary Mucinous Neoplasms (IPMNs), sarcomas, lymphomas or metastases) and (3) were lacking mental capacity. Participating carers had to be: (1) over 18 years of age, (2) individuals with mental capacity, 3. person of patient’s choice, whilst professional carers were excluded.

### Collection of baseline documentation

Baseline documentation collection consisted of note review shortly after the first research visit and recorded demographics (age and gender), significant co-morbidities (cardiac, respiratory, renal, hepatic and endocrine), smoking history and cancer stage on diagnostic CT scan (American Joint Committee of Cancer TNM classification) [31].

### Patient and carer questionnaires

Every patient completed the study questionnaire at the first research visit under the supervision of the research specialist nurse. Thereafter, patients were invited to complete a questionnaire at monthly intervals. This questionnaire included: (1) Eastern Cooperative Oncology Group (ECOG) medical performance status (Scale 0–5), (2) Visual analogue score (VAS) for pain, (3) current analgesic and non-analgesic drug use, (4) QoL questionnaires (EORTC 30 and EuroQol EQ-5D-5L) [32, 33] and (5) use of health care resources over the last month [34]. Carers completed the EQ-5D-5L questionnaire. They completed the first questionnaire during the first research visit and the same questionnaire monthly thereafter, on the same dates as the patient.

### Statistical analysis

Continuous variables were described using either means or medians according to their distributions. Categorical variables, including WHO medical performance status, were reported as frequencies and percentages. Kaplan-Meier analyses was conducted to estimate survival from diagnosis to the first opioid prescription and separately the survival time from pain onset. The exact onset of their pain is unknown as it preceded the completion of their first questionnaire. For the purpose of this survival analysis, it was assumed that their pain started at the time they completed their first questionnaire The differences in the characteristics between recruited participants and those who declined to participate were examined with Fisher’s exact test for the categorical variables (age and cancer stage) and Wilcoxon test for age. Difference in the EQ-5D-5L QoL scores over time were examined with the Friedman test for ordinal variables. All the parameters were analysed for each month from the entry to the study and up to six months. Some patients were recruited less than six months before the study completed and their outcomes were censored at the last date of follow-up.

### Health economic analysis

Earlier use of EUS-CPN is likely to be a more expensive treatment compared to opioids because of the infrastructure it requires (e.g. endoscopy equipment and highly trained staff). However, it may convey a higher health benefit through side-effect free pain control, and these health benefits may reduce other costs (e.g. fewer clinic attendances for pain control). Given uncertainty around the financial implications, a future trial could explore the impacts on quality life and the associated costs through a health economic analysis (specifically, a cost-utility analysis). If early use of EUS-CPN is beneficial and leads to reduced costs to the health system, it would be preferred to standard care (in health economic terms this is ‘domination’ [35]). However, should costs of EUS-CPN be greater, an ‘incremental cost-effectiveness ratio’ (ICER) is calculated by dividing the difference in total costs (incremental cost) by the difference in the chosen measure of health outcome or effect (incremental effect) to provide a ratio of ‘extra cost per extra unit of health effect’ [36]. Use of a QoL measure such as the EuroQol EQ-5D-5L allows utilities to be calculated, from which the ‘health effect’ can be quantified as quality adjusted life years (QALYs) which are “designed to combine the impact of gains in QoL and in quantity of life (i.e. life expectancy) associated with an intervention” [37]. Thus, if early EUS-CPN use leads to better outcomes but increased costs, the ICER can be compared to a pre-determined threshold to determine if the benefits are considered cost-effective: for example, NICE generally considers the threshold to be between £20–30K/QALY [38]. Accordingly, the feasibility of a health economic (cost-utility) analysis in a future trial is determined based on the feasibility of data collection to estimate resource use (and thus costs to the care system) and QoL. A study-specific health care cost questionnaire was adapted from the UK Cancer Costs Questionnaire (UKCC) Version 2.0 [34]. This questionnaire asks patients about their use of NHS, personal social services and “out of pocket” expenses (travel costs, parking and others) in the last month. Associated costs to the NHS and PSS (the NICE preferred costing perspective [38]) of this resource use were determined from Personal Social Services Research Unit’s (PSSRU’s) “Unit Costs of Health and Social Care 2020” [39] and NHS Reference costs [40]. The costing year was 2020, the latest for which costing resources were available at time of analysis (NHS reference costs were adjusted to 2020 values through appropriate use of the NHS cost inflation index (NHSCII)) [39]. QoL utilities were calculated from the EQ-5D-5L value set, using the value set for England [41]. Where a patient was known to have died, we ascribed them a utility value of 0 for subsequent QoL assessments. QALYs were calculated from the utilities by calculating the area under the curve with linear interpolation [42]. Completion rates of the resource use questionnaire and QoL measures were used to gauge the feasibility of a future economic evaluation. Patterns of missingness and feedback to data collectors were considered to see if they suggested questionnaire refinements that might optimise future data collection.

### Sample size considerations

A formal sample size calculation was not needed as this was an observational study to plan a future definitive randomised trial of early EUS-CPN vs standard care: as such, it did not have a particular primary outcome. However, based on cancer registry data, a total of approximately 90 patients in NNUH and JPUH are diagnosed with pancreatic cancer over an 18-month period, our intended duration of recruitment. Assuming that 25–30% of the patients would be ineligible on the basis of the poor general health or decline participation based on their choice, we aimed to recruit 65 patients.

### Patient and public involvement

Pancreatic Cancer UK information and Norfolk Together Against Cancer Organisation were actively involved in the design of BAC-PAC study. The groups revised the questionnaires and gave advice about the content and length, they made recommendations on when potential participants should be approached and were strong advocates for carer involvement.

### Funding

The NIHR Research and Design Service of East of England contributed to study design [43]. This study was funded by NIHR Research Capability Funding (RCN) and the NIHR Research for Patient Benefit (RfPB) scheme (reference number: PB-PG-0817-20028).

## Results

### Recruitment

**Fig. 2:**
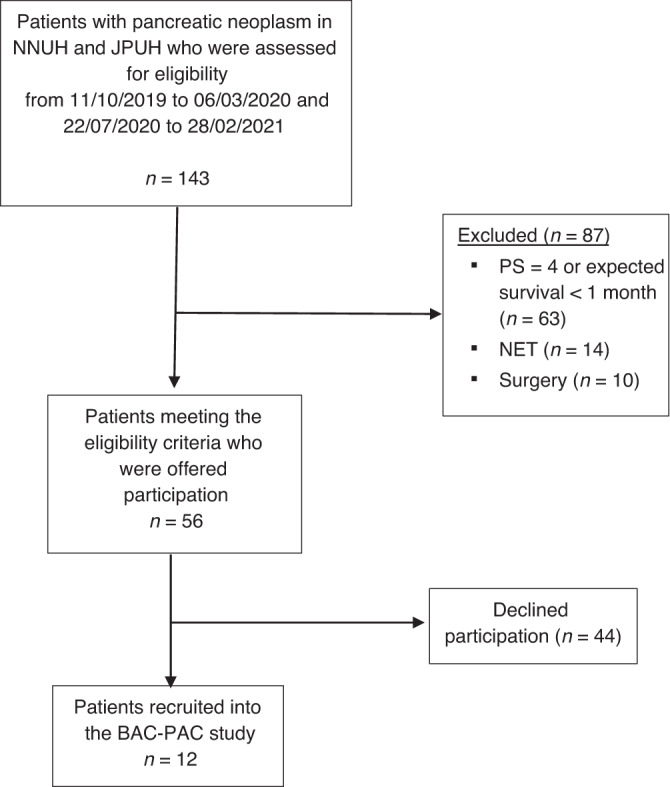
STROBE flow chart of the study participants.

During the recruitment period, from October 11th 2019 to March 6th 2020 and from July 22nd 2020 to February 28th 2021, 143 patients were diagnosed with pancreatic cancer and assessed for eligibility (Fig. 2). In total, 87 (61%) patients were excluded due to not satisfying eligibility criteria (63 (44%) were excluded on the basis of a very poor medical performance status and limited expected survival, 14 (10%) with NETs and 10 (7%) who underwent surgery). The remaining 56 patients were offered participation, of whom 12 (21%) consented.

### Demographics and clinical characteristics

Demographics and clinical characteristics of recruited patients are summarised in Table 1. Mean age at diagnosis was 71 (SD 9.9) years. Eight (66.7%) of the respondents were male. Out of the 12 patients, one (8.3%) had stage II, six (50%) had stage III and five (41.7%) had stage IV cancer. Chemotherapy was administered in nine (75%) of the participants. Median survival was 5.9 (IQR 4.8–11.0) months. Mean time from diagnosis to the first questionnaire completion was 39 (SD 16) days.

**Table 1:** Demographic and clinical characteristics of recruited patients.

Number of recruited patients	12
Number of recruited carers	8
Patients’ Age in years (mean, SD)	71 (9.9)
Male patients (*n*, %)	8 (66.7%)
Time from diagnosis to the first questionnaire completion in days (mean, SD)	39 (16)
Cancer Stage (*n*, %)	
II	1 (8.3%)
III	6 (50.0%)
IV	5 (41.7%)
Chemotherapy	9 (75.0%)
Survival in months (median, IQR)	5.9 (4.8–11.0)
Major Co-morbidities	
Cardiac	6 (50%)
Respiratory	3 (25%)
Hepatologic	1 (8.3%)
Renal	5 (45.5%)
Diabetes	3 (25%)
Smoking History	
Non-smoker	9 (75%)
Ex-smoker	2 16.7%)
Current-smoker	1 (8.3%)

*SD* standard deviation, *IQR* inter-quartile range.

### Medical performance status at the pain onset

In total, seven (58%) out of the twelve of the respondents were affected by pain by the time of their entry to the study. Their performance status varied between 0 and 2. One patient developed pain at month two and had performance status of 0 and another one developed pain at month three with a performance status of 2. The remaining three patients did not report pain during follow-up. The medical performance status of those respondents who reported pain is detailed in appendix 1.

### Median survival after pain onset

Seven of the twelve (58%) patients reported pain requiring opioids by the time of entry to the study. Another two patients developed pain, one a month after their study entry and the other two months after their study entry. Overall, three patients out of nine (33%) who had pain were alive by the date of the study close. The total analysis time at risk was 39.1 months. The median survival from pain onset was 5.2 (IQR 2.46–5.9) months. A Kaplan-Meier plot is shown in appendix 2.

### Characteristics of participants versus those who declined participation

Of the 56 patients approached, 44 (79%) refused participation (Table 2). Although some differences in the proportion of males (54% vs 67%) and the cancer stage IV (65% vs 47%) were observed, these differences were not statistically significant. Overall, 19 (52%) of those who declined participation, reported doing so because of severe emotional distress, 12 (31%) were not interested in participating in research, whilst four (11%) had an initial intention to participate, but developed chemotherapy complications and decided against participation.

**Table 2:** Demographic and clinical characteristics of patients who declined participation to BAC-PAC.

	Non-participants	Participants	*p*-value
Number of patients	44	12	
Age in years (median, IQR)	72 (59.9–78.5)	75 (65.9–80.4)	0.604
Male patients (*n*, %)	23 (53.5%)	8 (66.7%)	0.516
Cancer Stage (*n*, %)			
II	5 (11.6%)	1 (8.3%)	
III	10 (23.3%)	6 (50.0%)	0.267
IV	28 (65.1%)	5 (41.7%)	

The observed differences did not reach statistical significance.

### The QoL of carers of pancreatic cancer patients

In total, eight out of the twelve patients (75%) participated in the study were accompanied by a carer. The number of the participating carers diminished as patients died or dropped out. The mean EQ-5D-5L scores for the first three months of the study follow up are displayed in appendix 3. The global health VAS score and the summary index score demonstrate a static impairment of the QoL throughout the first three months. The differences across the months were not statistically significant (*p* < 0.05).

### Questionnaire completion rates

Questionnaire completion at each time point was calculated as a proportion of those who were still alive and invited to complete questionnaires. Dropouts refer to those who did not return their questionnaires, although they were still alive. Some patients had a reduced follow up time, as they started participating three months before the study’s end date; their questionnaire completion was therefore censored. Overall, 33 (80%) questionnaires were returned from a total of 41 which were expected. The completion rates were 100% in the first two months and gradually reduced to 33% at six months. Missing questionnaires resulted from two out of the twelve patients who dropped out at month three. Their missing questionnaires account for 20% of the questionnaires expected to be returned. Completion rates at each time point are detailed in Table 3. When questionnaires were returned, completion of the different sections was very good: only one patient declined to complete medication use section.

**Table 3:** Questionnaire completion rates in BAC-PAC participants.

Month	1	2	3	4	5	6	Total
Expected questionnaires from alive patients (*n*)	12	10	8	4	4	3	41
Returned questionnaires (*n*)	12	10	6	2	2	1	33
Completion rate^a^ (%)	100%	100%	75%	50%	50%	33%	80%
Dropouts^b^ (*n*, %)	0 (0%)	0 (0%)	2 (25%)	2 (50%)	2 (50%)	2 (76%)	20%
Deceased patients (*n*)	0	2	3	4	4	4	-
Patients whose completion was censored due to end of study^c^ (*n*)	0	0	1	4	4	5	-

^a^Completion rate is estimated as the number of returned questionnaires divided by the number of alive patients expected to return a questionnaire at each time-point.

^b^Dropouts refer to the proportion of patients who did not return their questionnaire despite being alive and are calculated for each time point.

^c^The completion rate was censored for patients who were recruited less than six months from the end of the study.

### Time from diagnosis to first opioid prescription

In total, seven (7/12, 58%) patients reported pain by the time of study entry. Of the remaining five patients, one reported pain one month after their entry, another one after two months from their entry and three patients (25%) did not develop pain during follow-up. The median time from study entry to pain onset could not be calculated, as more than 50% of the patients experienced the pain before the study entry. A Kaplan-Meier plot is shown in Appendix 4.

### The mean/median QoL, abdominal pain score and opioid dose

Analysis was limited to the first three months of follow-up, as beyond this point data were available for two or fewer patients. The EQ-5D-5L scores showed a gradual impairment of the global health VAS score, however the differences between month one, two and three were not statistically significant (*p* = 0.185) (Appendix 5). All the other elements of the EQ-5D-5L (mobility, self-care, usual activities, pain/discomfort, anxiety/depression) as well as the summary index score were relatively static. Analysis of the EORTC-QLQ30 score also demonstrated stable impairment of all the functioning scales (role, emotional, cognitive, social and cognitive) (Appendix 6). A relatively static impairment was also noted for fatigue, dyspnoea, diarrhoea and appetite loss. In contrast, pain, constipation and insomnia showed a trend for improvement. Nausea was the only symptom with a trajectory of deterioration. Financial difficulties remained zero throughout. The median VAS pain score was 2.9 (IQR 0.8 to 5.1) and 1.7 (IQR 1.0 to 1.9) at month one and two, respectively. The median daily morphine dose equivalents were 36 (IQR 20 to 48) at month one and 28 (IQR 6.8 to 70) at month two (Table 4). Only one out of the six (17%) respondents reported pain in month three.

**Table 4:** Visual analogue pain scores and morphine dose equivalents.

Month	1	2	3	4	5	6
Number of patients completing questionnaires (*n*)	12	10	6	2	2	1
Number of patients reporting pain (*n*, %)	7 (58%)	5 (50%)	1 (17%)	0	0	0
Number of patients alive (*n*)	12	10	9	8	6	5
VAS score (Median, IQR)	2.9 (0.8–5.1)	1.7 (1.0–1.9)	7.8	N/A^a^	N/A^a^	N/A^a^
Morphine dose equivalent in mg (median, IQR)	36 (20–48)	28 (6.8–70)	78	N/A^a^	N/A^a^	N/A^a^

^a^After month three two data were available for two or less patients, hence descriptive statistics were not calculated.

### Health economic analysis

Analysis of the resource use identified 17 types of expenditure. The estimated unit costs per resource and the assumptions made for the estimation of those costs are shown in Appendix 7. The mean NHS and PSS expenditure per patient was estimated at £1491 per month. A detailed breakdown of the expenditure per resource is shown in Table 5. The estimated median QALY were 0.073 (IQR 0.062 to 0.076) between month one and two and dropped to a median of 0.054 (IQR 0.020 to 0.076) between month two and month three Table 6.

**Table 5:** Units of medical resource used and actual expenditure per resource.

	Month 1 (12 participants)			Month 2 (10 participants)			Mean expenditure over the first two months
	Units used	Total expenditure per resource	Average expenditure per patient	Units used	Total expenditure per resource	Average expenditure per patient	
Hospital-based resources							
Days in-hospital stay	21	£9,387	£782	11	£4,917	£492	£7,152
Non-elective attendance to A&E or similar	1	£382	£32	3	£1,146	£115	£764
Delivery of parenteral chemotherapy at first attendance	9	£2,768	£231	0	-	-	£1,384
Delivery of subsequent elements of a chemotherapy cycle	0	-	-	27	£2,663	£266	£1,331
Radiotherapy	0	-	-	5	£714	£71	£357
Consultant appointments	11	£655	£55	8	£476	£48	£565
Specialist nurse appointments	21	£525	£44	44	£1,100	£110	£813
Community-based resources							
GP appointments	7	£275	£23	2	£78	£8	£177
GP telephone call	2	£17	£1	6	£50	£5	£4
Primary care nurse appointment	6	£147	£12	4	£92	£9	£120
Primary care nurse home visits	5	£115	£10	0	-	-	£58
Dietician appointment	2	£50	£4	3	£75	£8	£63
Occupational health appointment	1	£25	£2	0	-	-	£13
Community equipment (stairlift)	1	£654	£55	0	-	-	£327
Appointments with other health professionals	4	£100	£8	3	£75	£8	£88
Total costs		£15,099	£1,258		£17,711	£1,771	£16,405*
Medical prescriptions	-	£384	£35	-	£208	£20	
Out of pocket expenses							
Travel for medical appointments (in miles)	887	£133	£11	1972	£296	£30	£214
Car parking expenditure	-	£46	£4	-	£72	£7	£59

*Mean expenditure per patient for the first two months was £1491.

**Table 6:** Table of QALYs per month.

Month intervals	1st to 2nd	2nd to 3rd	3rd to 4th	4th to 5th	5th to 6th
Total number of patients in the cohort (*n*)	12	12	12	12	12
Alive patients contributing utility values at the start and the end of the month (*n*)	10	6	2	2	1
Deceased patients at each time interval^a^ (*n*)	2	3	4	4	4
Patients with missing data due to dropouts (*n*)	0	2	2	2	2
Patients with censored data^b^ (*n*)	0	1	4	4	5
Quality Adjusted Life Years (QALY)^c^ (median, IQR)	0.073 (0.062–0.076)	0.054 (0.020–0.076)	0^3^ (0.000–0.033)	0^3^ (0.000–0.083)	0^3^ (0.000–0.083)

^a^Deceased patients contributed with “0” utilities at the end of the month they died and for the subsequent months.

^b^Some patients entered the study less than six months before its closure. Their utility values from three to six months were censored.

^c^As this represents a QALY score from a month, the maximum QALY, at full health would be 0.083 (e.g. 1/12).

A narrative assessment, in terms of completeness, relevance and quality of the collected data was undertaken based on informal feedback from patients and members of the research team involved in data collection. This revealed that patients’ pattern of medical resource use consists of elective attendances for planning, consent and delivery of chemotherapy treatments as well as non-elective attendances to emergency services. However, it also revealed that our data collection instrument was not specific enough to capture the purpose of patients’ elective and non-elective attendances and the specific hospital department involved and the medical activities that were undertaken during those. For example, the number of non-elective attendances were questioned but it did not specify if this was for a cancer-related or a general medical problem. Similarly, if a patient attended for a chemotherapy infusion, we did not capture whether they were seen by the consultant or the specialist nurse during the same event. Imprecisions as such may have lead to significant cost misestimations. In terms of the completeness, one patient did not complete his drug record because he felt it was too time-consuming as it consisted of many items.

## Discussion

Overall, twelve out of the 56 eligible patients (21%) were recruited to the BAC-PAC study. There was no statistically significant difference in the age, gender and cancer stage between the recruited participants and those who declined participation; however, a lack of statistical significance may reflect the small sample size. Questionnaire completion rate was 80%. Completeness of the provided data was high overall. The medical performance status of those in pain varied between 0 and 2. The median survival from pain onset was 5.2 (IQR 2.5–5.9) months. Seven out of the twelve patients (58%) reported pain at baseline and another two developed in the subsequent months. Consequently, the median time from diagnosis to the pain onset could not be assessed; less than 50% of patients who were pain-free at diagnosis develop pain upon their entry to the study. The QoL was consistently impaired in the first three months based on the functioning scales of EORTC-QLQ30 and the summary index score deriving from the ED-5Q-5L. The median VAS pain score was 2.9 (IQR 0.8–5.1) at month one and 1.7 (IQR 1.0–1.9) at month two. The median daily morphine dose equivalents were 36 (IQR 20–48) and 28 (IQR 6.8–70) at months one and two, respectively. In total, eight out of twelve carers (75%) participated in the study. Their QoL was impaired based on the EQ-5D-5L QoL questionnaire. Overall, 17 different types of expenditure were identified, including hospital-based care, community-based care, medical prescription costs and “out of pocket” expenses. The mean NHS and PSS expenditure per patient was £1491 per month. The estimated median QALY were 0.073 (IQR 0.062 to 0.076) between month one and two and dropped to a median of 0.054 (IQR 0.020 to 0.076) between month two and month three. The health economic data collection instrument needs to be more specific about the purpose of the attendances and the specific activities undertaken during those and supplemented with medical record review.

Assessment of study objectives was limited by poor recruitment. This was the result of two waves of the COVID-19 pandemic but also lower than expected recruitment from eligible patients. This resulted in imprecise estimates for most objectives. We intended to record performance status at the time of pain onset, so that we could estimate how fitness for endoscopy may affect eligibility for early EUS-CPN in a future trial. The medical performance status of those with pain ranged from normal (performance status 0) to mildly impaired (performance status 2) and therefore, these patients’ general health should not preclude EUS-CPN. We showed that the median survival from pain onset is 5.2 (IQR 2.46–5.9) months. This is likely to be an underestimate, given that patients were typically recruited six weeks after diagnosis and onset of pain preceded this time point in the majority of them (7/9). Nevertheless, this is a meaningful period of survival time (i.e. at least three months) and they could potentially benefit from an early EUS-CPN. However, in view of limited study recruitment, it is unclear if this estimated survival can be generalised to all patients with inoperable pancreatic cancer. We aimed to compare the characteristics of those who participated versus those who refused, to evaluate the generalisability of our results. Age, gender and cancer stage were numerically similar and there were no statistically significant differences between the two groups. This is important as recruitment to a prospective observational study is likely to differ to that of a randomised controlled trial (participants may derive direct benefit if allocated to the intervention which may alter decisions around participation). We therefore have some indirect evidence to conclude that inferences from this research could be applied to a future trial.

We hypothesized that carer QoL declines as a result of patients’ uncontrolled pain and we aimed to explore if QoL in carers could be a secondary outcome in a future trial. Indeed, aspects of their life, such as mood, ability to attend usual activities and global health were adversely affected. However, QoL is multidimensional, and it is unclear whether improving pain when all other negative cancer consequences persist (reduced survival, frequent chemotherapy complications, cachexia etc) can produce any detectable QoL benefits for carers in the context of a clinical study. To determine this, the effect of early EUS-CPN on domains of QoL would need to be evaluated as a secondary outcome in a future RCT. The questionnaire completion rate was 100% in the first two months of follow-up, however completion rates fell to 75% by month three and continued to decline in subsequent months. Informal feedback from participants revealed that the burden of study activities was prohibitive for their adherence to follow-up. Consideration for this in a future study is needed, for example, questionnaires could be completed jointly with members of the research team (rather than self-reporting) and ensure only essential data collected, but also that routine sources of data and extraction from notes are used as primary sources.

This study intended to measure several fundamental parameters to inform the logistics of a future trial. Firstly, recording the time from diagnosis to the first opioid prescription was considered an important element, as this is when randomisation in a future trial could happen. Herein we showed that most patients presented with pain and therefore they would be randomised soon after diagnosis. Moreover, we calculated the descriptive statistics for the QoL scores, VAS pain scores and daily morphine consumption, one of which could reasonably serve as the primary outcome of a future trial. However, given the small sample it is unlikely these estimates can be relied upon and further assumptions are likely to be required in order to plan and design a future trial (particularly estimates of recruitment, retention and parameters on which to base a sample size calculation).

We also explored the feasibility of collecting data to inform a future cost-utility analysis, which would inform the cost-effectiveness of the different pain management alternatives. Costs would be based on the resource use recorded on the questionnaire: the corresponding sections were completed relatively well, but contributed to patient burden. Where possibly, a future trial may need to rely on collection from notes and service records to reduce patient burden and hopefully minimise drop-out. Completion of the EQ-5D-5L, from which utilities, and subsequently QALYs, are calculated for the cost-utility analysis were relatively good, suggesting its use is feasible.

Two previous retrospective cohort studies have comprehensively assessed the epidemiological characteristics of pain in patients with inoperable pancreatic cancer [4, 5]. In those the prevalence of pain was estimated between 58–78% [4, 5]. Our estimate of 58%, falls within the lower end of this range and this is possibly because our study may not have included patients with the most severe pain who could not engage with study procedures, such as questionnaire completion. The previously reported mean daily opioid dose of 55.9 (SD 53.8) mg at diagnosis [4] is higher than our estimate of 39 (SD 25) mg (our results reported median values to reflect their skewed distribution, but here we report mean values to facilitate direct comparison with previous literature). This discrepancy, similar to the prevalence of pain, probably reflects that our study, due to the method of data collection, recruited patients with preserved general health who are less likely to use high dose opioids. The same previous paper estimated a mean period of 3.2 (SD 7.7) months from diagnosis to the first opioid prescription [4]. In our study this figure was not measurable, as 58% of the patients already had pain by the time they entered the study, so a median time is not informative. The mean survival time from the pain onset was 6.2 (SD 6.9) months which is similar to our findings (median survival 5.2, IQR 2.46 to 5.9).

Several studies have reported QoL scores in pancreatic cancer. These studies were conducted either for questionnaire validation or measuring QoL outcomes in the context of chemotherapy. We have chosen four of them for comparison with our results, based on their relevance, rigor and contemporality [44–48]. Two studies have reported the EORTC-QLQ30 in patients with inoperable pancreatic cancer [44, 45]. One prospective cohort study of 116 patients undergoing chemotherapy reported EORTC-QLQ30 global health scores for month one, two and three of 50.8%, 46.8% and 48.4%, respectively (SD not provided) [44]. These were broadly similar to our results, which were 53% (SD 22), 58% (SD 22) and 57% (SD 28) at the same time points, considering that the EORTC group defines clinically meaningful results as any difference in excess of ≥10% [49]. EORTC pain, fatigue, sleep disturbance and loss of appetite scores were the most affected quality of life components, with a score around 40 (scale 0 to 100), which are very similar to our results. Four studies, one from United States, Canada, Norway and Japan, reported EQ-5D-5L scores; the summary index ranged from 0.62 to 0.82 at month one and 0.64 to 0.69 at month two [45–48]. These values are lower than our estimates; 0.86 (0.12), 0.87 (0.12) at months one and two. Three reasons may explain this disparity. Firstly, the EQ-5D-5L is a generic instrument, not specifically designed to capture cancer-related impairment. Secondly, it is validated against societal preferences for given health states which, by definition, are variable among ethnicities [32]. Thirdly, only 41% of our participants had metastatic disease, whilst in the above studies this percentage was 70% and above.

The poor recruitment rate in observational studies targeting patients with newly diagnosed pancreatic cancer is not unique to our study; a multi-centre, questionnaire-based, prospective observational study, aiming to investigate the predictive value of common presenting symptoms (jaundice, nausea, weight loss etc) with the risk of pancreatic cancer in patients newly referred from the primary care to the relevant cancer pathway in seven UK and Australian hospitals, recruited only 24% of the eligible patient population [50]. Similar low participation rates were observed in studies with a prospective design in patients with lung and colorectal cancer [51, 52]. This is likely to be attributed to the psychological and physical effects a cancer diagnosis places on patients.

To the best of our knowledge, there is no previous literature with which to compare on the performance. Overall, our results, including prevalence of pain, opioid doses and QoL, are indicative of patients with a better general health in comparison to those in previous studies; this suggests that questionnaire self-completion is probably prohibitive for the participation of the more unwell patients.

Several methodological strengths were observed in the BAC-PAC study. It is unlikely that eligible cases were missed: there was a systematic screening on a weekly basis of the cancer registry, review of the MDT notes and other medical records and liaison with the direct clinical team to ensure all cases were identified and eligibility was accurately evaluated. The prospective design of this study enabled to capture real-time patient reported outcomes. In addition, the conduct of this study, informed by PPI, sought to minimise burden and impact on the very vulnerable patient group. For example, there were occasions understandably when patients and their carers were struggling to process their diagnosis and prognosis due to emotional distress. In those cases, the time to approach patients was carefully considered in consultation with the direct care team. Similarly, to avoid intrusiveness, contacts with patients were discontinued when patients were repeatedly not returning their questionnaires despite gentle reminders from the research team. Although these considerations impacted recruitment, they highlight the fragile psychology of this patient group an important consideration for any future trial of early EUS-CPN. On the other hand, the study suffered several limitations mainly related to the small number of participants, limiting assessment of objectives with resulting imprecision. Involvement of other centres was attempted to improve poor recruitment; unfortunately this coincided with the second wave of the COVID-19 pandemic and could not be progressed. Unfortunately, the use of self-completed questionnaires (which were informally viewed as burdensome) likely had a negative impact on recruitment.

Our study, being exploratory in nature, has revealed important aspects relevant to a future trial of early EUS-CPN versus standard care. We have demonstrated that pain has a high prevalence among patients with inoperable pancreatic cancer; the need of opioids is frequently observed; QoL of both patients and carers is impaired, with pain being one of the contributors to this impairment. All these findings support the rationale for a future trial of early EUS-CPN. In addition, the patients’ performance status and the survival after the pain onset lend further support for feasibility. However, given the limitations of this study, further information is required to determine the specific design of a future trial. Robust estimates of the prevalence of pain, opiate burden and related adverse events are needed over time in this population to better inform justification. Another finding with implications for a future trial were low recruitment rates, and barriers to recruitment would need to be addressed. Attention to recruitment and retention procedures in a trial are important and will require extensive PPI to develop and implement. Furthermore, informal feedback from patients and carers suggested that questionnaire completion may be perceived as a laborious task during one’s terminal illness. Therefore, greater reliance on routinely collected clinical data is needed, to minimise the burden placed on patients. For example, medication use could be ascertained using routinely collected data from primary and secondary care. The conduct of a health economic analysis is feasible, however refinement of the data collection instrument is needed to primarily use medical records and related sources of information, to reduce participant burden. Finally, the small number of patients who were eligible for the BAC-PAC and the even smaller of those who suffered from pain, indicates that a future multi-centre feasibility trial is required to ensure adequate recruitment for suitable statistical power.

Pain is prevalent in 58% of the patients with advanced pancreatic cancer at diagnosis. Survival is likely sufficient (median survival time: 5.2, IQR 2.46 to 5.9) months) to permit endoscopic analgesia. However, further research is needed to provide more precise estimates of the prevalence of pain, the doses of opioids and survival to improve the assessment of the justification and planning of a future trial. Careful attention to enablers and barriers to recruitment need to be considered in this patient population.

## Appendix 1

Medical performance status of patients reporting pain.

**Table:**

Months	1	2	3	4	5	6
Patients at risk (*n*)	12	10	8	4	4	3
Patients returning questionnaires (*n*)	12	10	6	2	2	1
Patients reporting pain (*n*, %)	7 (58%)	5 (50%)	1 (17%)	0	0	0
Patients whose completion was censored^1^ due to end of study (*n*)	0	0	1	4	4	5
Performance status^2^ (*n*, %)						
0	1 (14%)	3 (60%)	-	-	-	-
1	3 (43%)	1 (20%)	-	-	-	-
2	3 (43%)	1 (20%)	1	-	-	-
3 or 4	-	-	-	-	-	-

^1^Censored are the patients whose follow up was ceased due to end of the study.

^2^The displayed performance status refers only to patients with pain, who therefore would be EUS-CPN candidates, if a clinical trial was running.

## Appendix 2

Figure 23. Kaplan-Meier plot demonstrating mortality over time since pain onset. Overall, nine out of the twelve patients (75%) developed pain either at baseline or follow-up.

## Appendix 3

QoL scores in carers calculated based on the EQ-5D-5L QoL questionnaire.

**Table:**

	Month 1	Month 2	Month 3	*p*-value
Number of participating carers (*n*)	8	6	5	
Mobility (mean, SD)	1.13 (0.35)	1.50 (0.55)	1.40 (0.54)	0.532
Self-care (mean, SD)	1.13 (0.35)	1.00 (0.00)	1.00 (0.00)	1.00
Usual activities (mean, SD)	1.50 (0.92)	1.33 (0.51)	1.20 (0.44)	0.494
Pain/discomfort (mean, SD)	1.50 (1.00)	1.50 (0.83)	1.40 (0.54)	0.494
Anxiety/depression (mean, SD)	2.00 (0.53)	1.83 (0.75)	1.60 (0.54)	0.187
Summary Index score (mean, SD)	0.86 (0.16)	0.87 (0.13)	0.89 (0.11)	0.098
Global health VAS score (mean, SD)	76.9 (25.5)	88.3 (10.3)	84.0 (13.8)	0.127

Figures in the five dimensions (mobility, self-care, usual activities, pain/discomfort and anxiety/depression) represent mean values in a 5-point scale from 1 to 5 where 1 is perfect health and 5 highest degree of impairment.

Only one carer participated beyond month three, therefore descriptive statistics were not calculated.

**Table 10:** Medical performance status of patients reporting pain.

Months	1	2	3	4	5	6
Patients at risk (*n*)	12	10	8	4	4	3
Patients returning questionnaires (*n*)	12	10	6	2	2	1
Patients reporting pain (*n*, %)	7 (58%)	5 (50%)	1 (17%)	0	0	0
Patients whose completion was censored^a^ due to end of study (*n*)	0	0	1	4	4	5
Performance status^b^ (*n*, %)						
0	1 (14%)	3 (60%)	-	-	-	-
1	3 (43%)	1 (20%)	-	-	-	-
2	3 (43%)	1 (20%)	1	-	-	-
3 or 4	-	-	-	-	-	-

^a^Censored are the patients whose follow up was ceased due to end of the study.

^b^The displayed performance status refers only to patients with pain, who therefore would be EUS-CPN candidates, if a clinical trial was running.

## Appendix 4

Kaplan-Meier plot: time from study entry to pain onset. Five out of twelve (42%) patients were pain-free at their entry, of whom two (40%) developed pain in the first two months.

## Appendix 5

EQ-5D-5L QoL scores for patients.

**Table:**

	Month 1	Month 2	Month 3	*p*-value
Number of participating patients (*n*)	12	10	6	
Mobility (mean, SD)	1.4 (0.79)	1.3 (0.48)	1.8 (0.83)	0.237
Self-care (mean, SD)	1.1 (0.29)	1 (0)	1.2 (0.45)	0.955
Usual activities (mean, SD)	2.3 (1.21)	1.8 (0.92)	2.6 (1.52)	0.209
Pain/discomfort (mean, SD)	1.9 (0.90)	1.9 (0.88)	2.0 (1.0)	0.143
Anxiety/depression (mean, SD)	1.4 (0.79)	1.4 (0.51)	1.8 (0.83)	0.129
EQ-5D-5L index value (mean, SD)	0.86 (0.12)	0.87 (0.12)	0.78 (0.20)	0.102
Global health VAS score (mean, SD)	71.3 (21.3)	67 (18.2)	51 (15.1)	0.185

Only two patients participated beyond month three, therefore descriptive statistics were not calculated.

## Appendix 6

Table of the function scores and symptoms of the EORTC-QLQ30 over time.

**Table:**

Month	1	2	3
Participants	*n* = 12	*n* = 10	*n* = 6
	Mean (SD)	Mean (SD)	Mean (SD)
Global health status^a^	53 (22.0)	58 (22.0)	56.7(27.9)
Physical functioning^a^	74 (21.7)	78 (21.8)	70.7 (29.3)
Role functioning^a^	72 (30.4)	78 (23.6)	63.3 (44.7)
Emotional functioning^a^	69 (22.6)	70 (24.3)	71.7 (32.6)
Cognitive functioning^a^	81 (30.0)	85 (14.6)	83.3 (23.6)
Social functioning^a^	64 (24.4)	70 (24.6)	73.3 (25.4)
Fatigue^b^	44 (31.9)	43 (31.2)	55.6 (35.1)
Nausea and vomiting^b^	13 (22.6)	18 (21.4)	33.3 (23.6)
Pain^b^	33 (36.2)	18 (19.9)	13.3 (29.8)
Dyspnea^b^	19 (30.0)	20 (35.8)	20 (29.8)
Insomnia^b^	36 (30.0)	23 (22.5)	13.3 (29.8)
Appetite loss^b^	50 (41.4)	53 (39.1)	53.3 (29.8)
Constipation^b^	39 (37.2)	10 (16.1)	6.7 (14.9)
Diarrhea^b^	36 (38.8)	33 (47.1)	46.7 (38.0)
Financial difficulties^b^	0 (0)	0 (0)	0 (0)

^a^Functioning scores in EORTC-QLQ30 are scaled from 0 to 100. 0 represents worst possible functioning and 100 represents perfect health.

^b^Symptom scores in EORTC-QLQ30 are scaled from 0 to 100. In contrast to the functioning scores, 0 represents absence of a symptom whilst 100 represents the highest level of impairment in the QoL due to the examined symptom.

**Table 15:** Table of the function scores and symptoms of the EORTC-QLQ30 over time.

Month	1	2	3
Participants	*n* = 12	*n* = 10	*n* = 6
	Mean (SD)	Mean (SD)	Mean (SD)
Global health status^a^	53 (22.0)	58 (22.0)	56.7(27.9)
Physical functioning^a^	74 (21.7)	78 (21.8)	70.7 (29.3)
Role functioning^a^	72 (30.4)	78 (23.6)	63.3 (44.7)
Emotional functioning^a^	69 (22.6)	70 (24.3)	71.7 (32.6)
Cognitive functioning^a^	81 (30.0)	85 (14.6)	83.3 (23.6)
Social functioning^a^	64 (24.4)	70 (24.6)	73.3 (25.4)
Fatigue^b^	44 (31.9)	43 (31.2)	55.6 (35.1)
Nausea and vomiting^b^	13 (22.6)	18 (21.4)	33.3 (23.6)
Pain^b^	33 (36.2)	18 (19.9)	13.3 (29.8)
Dyspnea^b^	19 (30.0)	20 (35.8)	20 (29.8)
Insomnia^b^	36 (30.0)	23 (22.5)	13.3 (29.8)
Appetite loss^b^	50 (41.4)	53 (39.1)	53.3 (29.8)
Constipation^b^	39 (37.2)	10 (16.1)	6.7 (14.9)
Diarrhea^b^	36 (38.8)	33 (47.1)	46.7 (38.0)
Financial difficulties^b^	0 (0)	0 (0)	0 (0)

^a^Functioning scores in EORTC-QLQ30 are scaled from 0 to 100. 0 represents worst possible functioning and 100 represents perfect health.

^b^Symptom scores in EORTC-QLQ30 are scaled from 0 to 100. In contrast to the functioning scores, 0 represents absence of a symptom whilst 100 represents the highest level of impairment in the QoL due to the examined symptom.

## Appendix 7

Unit costs per medical resource or other health-related expenditure.

**Table:**

Resource	Unit Cost	Reference	Assumption
Hospital-based resources			
Hospital admission	£ 447.00	PSSRU (2020) section 7.1, NHS reference costs for hospital services, p87.	Patients receive palliative care and chemotherapy side-effect treatments
Non-elective attendance to A&E or similar	£ 382.00	National schedule of NHS cost 2018/2019, code SB97Z, tab: non-elective short stay (NES).	A&E attendances are be related to chemotherapy or procedure complications or due to poorly controlled symptoms attributed to cancer progression. Co-incidental illnesses rarely led to admission in patients with pancreatic cancer, hence are not considered in the costings [53, 54].
Delivery of parenteral chemotherapy at first attendance	£ 307.58	National schedule of NHS cost 2018/2019, code SB13Z.	Costs reflect delivery of complex chemotherapeutic schemes, such as FOLFIRINOX, but not single agent chemotherapy such as gemcitabine or capecitabine. Costs adjusted for inflation.
Delivery of subsequent elements of a chemotherapy cycle	£ 332.83	National schedule of NHS cost 2018/2019, code SB15Z.	Costs reflect delivery of complex chemotherapeutic schemes, such as FOLFIRINOX, but not single agent chemotherapy such as gemcitabine or capecitabine. Costs adjusted for inflation.
Radiotherapy	£ 142.88	National schedule of NHS cost 2018/2019, code 800, tab: total outpatient attendance.	No assumptions made. Costs adjusted for inflation.
Consultant: medical	£ 59.50	PSSRU (2020) section 14, hospital-based doctors, p159.	Consultant cost per working hour £119. Appointment length 30 mins (incorporating administrative tasks).
Dietician appointment	£ 25.00	PSSRU (2020) section 12, hospital-based scientific and professional staff, p151	NHS band 6 dietician, with cost per working hour 50. Appointment length 30 mins (incorporating administrative tasks)
Occupational health appointment	£ 25.00	PSSRU (2020) section 12, hospital-based scientific and professional staff, p151	NHS band 6 occupational therapist, with cost per working hour 50. Appointment length 30 mins (incorporating administrative tasks)
Specialist nurse appointments	£ 25.00	PSSRU (2020) section 12, hospital-based nurses, p155.	NHS band 6 nurse, with cost per working hour £50. Appointment length 30 mins (incorporating administrative tasks)
Community-based resources			
GP surgery consultations	£ 39.23	PSSRU (2020) section 10.3b, community-based health care staff-general practitioner, p126.	This unit cost is calculated based on the average duration of GP contact per patient, lasting 9.22 minutes.
GP telephone call	£ 8.41	PSSRU (2020) section 10.4, the cost of online consultations, p128.	This unit cost represents telephone contacts for following up tests or treatments that were decided during a GP surgery consultation.
Primary care nurse appointment	£ 24.50	PSSRU (2020) section 10.1, the cost of online consultations-nurses, p123.	NHS band 6 nurse, with cost per working hour 49. Appointment length 30 mins (incorporating administrative tasks)
Primary care nurse home visits	£ 23.00	PSSRU (2020) section 10.2, GP practice nurse, p124.	NHS band 6 primary care nurse, with cost per working hour 42 plus an average of 10 miles stuff travel per visit. Appointment length 30 mins (incorporating administrative tasks)
Community equipment (stairlift)	£ 654.00	PSSRU (2020) section 7.3, equipment and adaptations, p90.	No assumptions made.
Medical prescriptions			
Medical prescriptions		British National Formulary, URL: https://bnf.nice.org.uk/drug/, accessed on: 05/08/2021.	Costs were estimated based on the medication use the patients recorded on their self-completed questionnaires.
“Out of pocket” expenses			
Travel for medical appointments (in miles)	£ 0.15	AA motor insurance company, mileage calculator, URL: https://www.theaa.com/driving/mileage-calculator.jsp, accessed on: 30/07/21	Approximate fuel cost £1.40 per litre and engine performance rate of 40 miles per gallon.
Car parking expenditure	n/a	Costs directly reported by patients on self-completed medical resource questionnaire	No assumptions made.
